# Genome-Wide Transcriptional Responses of *Mycobacterium* to Antibiotics

**DOI:** 10.3389/fmicb.2019.00249

**Published:** 2019-02-20

**Authors:** Julien Briffotaux, Shengyuan Liu, Brigitte Gicquel

**Affiliations:** ^1^Department of Tuberculosis Control and Prevention, Shenzhen Nanshan Center for Chronic Disease Control, Shenzhen, China; ^2^Emerging Bacterial Pathogens Unit, CAS Key Laboratory of Molecular Virology and Immunology, Institut Pasteur of Shanghai, Chinese Academy of Sciences, Shanghai, China; ^3^Mycobacterial Genetics Unit, Institut Pasteur, Paris, France

**Keywords:** *Mycobacterium*, antibiotics, microarrays, RNA-seq, transcriptome, tuberculosis

## Abstract

Antibiotics can stimulate or depress gene expression in bacteria. The analysis of transcriptional responses of *Mycobacterium* to antimycobacterial compounds has improved our understanding of the mode of action of various drug classes and the efficacy and effect of such compounds on the global metabolism of *Mycobacterium*. This approach can provide new insights for known antibiotics, for example those currently used for tuberculosis treatment, as well as help to identify the mode of action and predict the targets of new compounds identified by whole-cell screening assays. In addition, changes in gene expression profiles after antimycobacterial treatment can provide information about the adaptive ability of bacteria to escape the effects of antibiotics and allow monitoring of the physiology of the bacteria during treatment. Genome-wide expression profiling also makes it possible to pinpoint genes differentially expressed between drug sensitive *Mycobacterium* and multidrug-resistant clinical isolates. Finally, genes involved in adaptive responses and drug tolerance could become new targets for improving the efficacy of existing antibiotics.

## Introduction

Tuberculosis (TB) is an infectious disease that typically attacks the lungs, it is caused by the bacteria *Mycobacterium tuberculosis*. This infection has possibly been present in humans since the Neolithic period (Comas et al., 2013). Today, TB is a re-emerging disease and a major public health problem in both developing and developed countries. According to the WHO, 10.4 million people fell ill with TB and 1.4 million died from the disease in 2016 (WHO, 2017). This pathogen can invade the pulmonary alveoli, where it is engulfed by macrophages. The success of this pathogen is due to its ability to survive inside macrophages, evade host defense mechanisms, and manipulate phagosome maturation (Ehrt and Schnappinger, 2009).

Public hygiene and the discovery of streptomycin and para-aminosalicylic acid (PAS) in the 1940s advanced the treatment of TB. In order to improve the efficiency of the treatment, other molecules have been used in combination, such as isoniazid from 1952, ethambutol from the early 1960s, and rifampin from the 1970s (Murray et al., 2015). Current TB treatment requires patients to take a combination of four drugs, called first-line TB drugs: isoniazid (INH), rifampicin (RIF), ethambutol (EMB), and pyrazinamide (PZA), for 2 months (initial phase) and two of them (INH and RIF) for an additional 4 months (continuation phase). Although very efficient, such treatment is long and very demanding. In many settings, the absence of a sufficient healthcare infrastructure and shortage of antibiotics can promote the appearance of resistance. In recent decades, multidrug-resistant TB (MDR-TB), extremely drug-resistant TB (XDR-TB) strains have emerged (Almeida Da Silva and Palomino, 2011; Udwadia et al., 2012). Treating and curing drug-resistant TB is complicated and more than 12 anti-TB drugs can be used for second-line regimens. These drugs are used in varying combinations depending on the circumstances; they include the aminoglycosides (streptomycin, kanamycin, amikacin, and paromomycin), the fluoroquinolones (moxifloxacin, ciprofloxacin, ofloxacin, and levofloxacin), the rifamycins (rifampin, rifabutin, and rifapentine), the beta-lactams (imipenem, tebipenem, and amoxicillin-clavulanic acid), oxazolidinone (linezolid), riminophenazine (clofazimine), ethionamide, PAS, bedaquiline, and SQ109. The targets and drug resistance mechanisms of these compounds have been recently reviewed (Islam et al., 2017), as well as the state of current drug development for the treatment of TB (Hoagland et al., 2016; Tandon and Nath, 2017).

The emergence of resistant strains of *M. tuberculosis* highlights the need to discover and develop new antimycobacterial compounds and approaches. It is equally important to understand the mode of action of these molecules, their effect on the cell, and the mechanisms by which bacteria can develop resistance.

The study of the biology of *M. tuberculosis* has been facilitated during the last 20 years by the availability of genome sequencing and genetic tools that allow the deciphering of major, specific metabolic pathways. For example, genetic and biochemical approaches resulted in the discovery of genes carrying mutations that confer isoniazid, ethambutol, ethionamide, and pyrazinamide resistance (Palomino and Martin, 2014). Microbial whole-genome sequencing allows the rapid detection of antibiotic susceptibility and resistance by the identification of resistance mutations (Takiff and Feo, 2015). However, this approach provides no information about the physiological state of the *Mycobacterium* or antibiotic tolerance due to changes in the transcriptional profile. In addition to the acquired resistance caused by target mutations, several distinctive mechanisms of antimycobacterial resistance have been described (Nasiri et al., 2017): the prevention of access to the target due to impermeability of the mycobacterial cell wall, transport of antimycobacterial compounds out of the cell by efflux pumps, modification of antibiotics by mycobacterial enzymes, and the modulation of gene expression, all leading to antibiotic tolerance.

Antibiotics can affect bacteria at many levels in addition to their direct effects on the target. These include effects on their morphology, metabolism, gene expression, stress response, and mutation rate (Nonejuie et al., 2013; Mitosch and Bollenbach, 2014; Tsai et al., 2015). Moreover, *Mycobacterium* can tolerate antibiotics due to their ability to reduce their intracellular accumulation by increasing active efflux of these molecules (Poole, 2007; Balganesh et al., 2012).

New knowledge concerning metabolic changes and adaptive responses of *Mycobacterium* after antibiotic exposure would help us to better understand both the mechanism of action of the antibiotics and the mechanisms of antibiotic resistance. Understanding how antimycobacterial compounds kill bacteria and the cellular response of the bacteria to such compounds is crucial to improving the efficacy and reducing the cytotoxicity of these drugs.

Altering transcription and adjusting physiology are amongst the main mechanisms in the initiation of adaptive processes in a cell (Cases et al., 2003; Perez and Groisman, 2009; Brooks et al., 2011). In *Mycobacterium*, the transcriptional response to a perturbation, such as antibiotic exposure, nutrient starvation (Betts et al., 2002), or limited oxygen (Bacon et al., 2004; Voskuil et al., 2004), can be assessed by transcriptomic techniques, such as microarrays and RNA sequencing (RNA-seq). DNA microarrays (DNA biochip), invented in the 1990s, are a collection of nucleotide probes or complementary DNA (cDNA) fixed on the surface that can hybridize to RNA, making it possible to analyze gene expression levels. However, the more recent development of RNA-seq, a method in which RNA is purified and cDNAs directly sequenced, enables rapid analysis, offering more flexibility (Mutz et al., 2013). Real-time quantitative reverse transcriptase-PCR (RT-qRT-PCR) is used to confirm changes in gene expression for both microarrays and RNA-seq.

**FIGURE 1:**
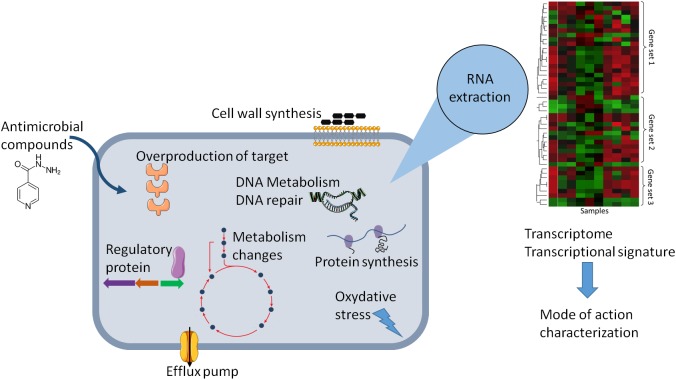
Schematic representation of the effect of antibiotic on the physiology of bacteria that can be explored by change in gene expression. Gene expression dynamic can reflect cellular response in the presence of antibiotic and can lead altered cellular state or adaptive responses and antibiotic tolerance. The transcriptional signature analysis allows to have new insights in the molecular mechanism of antimicrobial compounds or to predict the mode of action of uncharacterized antimicrobial compounds.

This review provides an analysis of published transcriptional-profiling data of *Mycobacterium* exposed to various antimycobacterial compounds (Table 1). Overall, theses microarrays or RNA-seq analyses can be used in various ways, depending on the question asked. It can be used to investigate changes in the gene-expression profile of bacteria following antibiotic exposure compared to that of untreated cells (Figure 1), the gene-expression profile of mutants relative to that of wild type cells treated with an antibiotic, or transcriptional profiles of clinical strains, especially MDR strains. Genome-wide expression profiles facilitate the characterization of both the mechanisms of action and the mechanisms of resistance to antimicrobial agents.

**Table 1:** Chronology of publications cited in this review on transcriptomic profiling by microarray (ma) or RNA-seq (rs) after anti-bacterial compound treatment.

*Mycobacterium species*	Drug			Reference
*M. tuberculosis*	Isoniazid	ma	wt	Wilson et al., 1999
*M. tuberculosis*	Isoniazid, triclosan	ma	wt	Betts et al., 2003
*M. tuberculosis*	Isoniazid, isoxyl	ma	wt	Waddell et al., 2004
*M. tuberculosis*	Various metabolism inhibitors	ma	wt	Boshoff et al., 2004
*M. tuberculosis*	Capreomycin	ma	wt	Fu and Shinnick, 2007
*M. tuberculosis*	Ciprofloxacin	ma	wt	O’Sullivan et al., 2008
*M. tuberculosis*	Vancomycin	ma	wt	Provvedi et al., 2009
*M. tuberculosis*	PA-824	ma	wt	Manjunatha et al., 2009
*M. tuberculosis*	Chelerythrine	ma	wt	Liang et al., 2011
*M. tuberculosis*	Linezolid	ma	wt	Liang et al., 2012
*M. tuberculosis*	Rifampicin	ma	m	de Knegt et al., 2013
*M. tuberculosis*	Meropenem	rs	wt	Lun et al., 2014
*M. smegmatis*	Bedaquiline	ma	wt	Hards et al., 2015
*M. smegmatis*	Isoniazid	ma	m	Padiadpu et al., 2015
*M. tuberculosis*	Isoniazid	ma	m	Yu et al., 2015
*M. tuberculosis*	Bedaquiline	ma	wt	Peterson et al., 2016
*M. tuberculosis*	Kanamycin	rs	wt	Habib et al., 2017
*M. tuberculosis*	Amoxicillin, clavulanate	ma	wt	Mishra et al., 2017
*M. tuberculosis*	AX-35	rs	wt	Foo et al., 2018
*M. tuberculosis*	Rifampicin	rs	m	Xu et al., 2018


*It also indicates if the work was done with wild type (wt) strain or with mutant strains (m) resistant to antibiotic.*

The deregulated genes in response to antibiotic treatment in *Mycobacterium* based on their fold expression, reported in most of the papers in this review, are analyzed and categorized into 10 functional classes: (1) virulence, detoxification, and adaptation; (2) lipid metabolism; (3) information pathways; (4) cell wall and cell processes; (5) insertion sequences and phages; (6) PE and PPE proteins; (7) intermediary metabolism and respiration; (8) proteins with unknown function; (9) regulatory proteins; and (10) conserved hypothetical proteins. From these data, it is possible to propose a role for certain genes in the response and adaptation to a given drug and a transcriptional signature for the drug, possibly highlighting transcriptional regulators and regulatory networks involved in the response.

## Isoniazid Induced Changes in Gene Expression

The first study to investigate changes in gene expression after antibiotic treatment of *Mycobacterium* was published in 1999 (Wilson et al., 1999). In this study, DNA microarrays were used to monitor gene-expression changes in response to isoniazid, one of the most active antibiotics used in TB treatment. Isoniazid is a prodrug and must be activated by a catalase-peroxidase (KatG) of *Mycobacterium*, to form an isonicotinic acyl anion, which reacts with NAD^+^ and binds to the active site of an enoyl-acyl carrier protein reductase, InhA (Rawat et al., 2003). This process prevents the synthesis of mycolic acids, components of the mycobacterial cell wall, thus resulting in death of the cells. Among several isoniazid induced genes, the authors highlighted the induction of five genes organized as an operon (*fabD-acpM-kasA-kasB-accD6*) and encoding type II fatty acid synthase enzymes (FAS-II), involved in the production of membrane phospholipids, as well as the gene encoding FbpC (Ag85C), an enzyme involved in mycolic acid transfer during cell-wall formation. It is possible that these genes are upregulated by a feedback mechanism which responds to the decrease of mature mycolates inside the cell. *inhA* is not induced in response to isoniazid treatment, nevertheless, by using *M. tuberculosis* strains with multicopy *inhA* or *kasAB* plasmids, it has been observed that the overexpression of *inhA*, but not *kasA*, confers resistance to isoniazide (Larsen et al., 2002).

The other genes do not appear to have any link with the biosynthesis of these fatty acids, but may be induced because of the toxic effects of isoniazid. Moreover, upregulation of the expression of the gene *efpA*, which encodes a major facilitator superfamily (MFS) efflux pump, as well as the upregulation of *iniA*, which encodes putative pump component, could participate in intrinsic resistance to isoniazid, as has been reported in other studies (Colangeli et al., 2005; Gupta et al., 2010; Li et al., 2015; Narang et al., 2017). *iniA* is upregulated along with *iniB* and *iniC*, these genes are indeed organized in operon under the control of *iniBAC* promoter and upregulated by various cell envelope inhibitor (Alland et al., 1998, 2000).

Another study investigated gene expression changes in *M. tuberculosis* following exposure to isoniazid, as well as thiolactomycin and triclosan (Betts et al., 2003). All three drugs are inhibitors of mycolic acid biosynthesis, but have different modes of action. The authors compared the response to these three drugs and proposed a transcriptional profile model that allows discrimination between *M. tuberculosis* treated with isoniazid, thiolactomycin, or triclosan. The model can then be used to specify the mode of action of uncharacterized mycolic acid biosynthesis inhibitors or characterize new InhA inhibitors during the drug discovery and development process. The gene expression profiles induced by isoniazid treatment were also compared to those induced by thiocarlide (isoxyl), a mycolic acid biosynthesis inhibitor and tetrahydrolipstatin (Waddell et al., 2004).

Isoniazid is less active in slowly or non-replicating mycobacteria. A study investigated isoniazid-regulated genes in drug-treated dormant *M. tuberculosis* and katG-deficient, isoniazid-resistant *M. tuberculosis* (Karakousis et al., 2008). Nutrient starvation and progressive oxygen depletion were used as *in vitro* models of dormancy. The transcriptional profile reflecting the mechanism of action of isoniazid was not found under these conditions nor in the katG mutant, in contrast to wild type *M. tuberculosis* after isoniazid exposure. The absence of the characteristic isoniazid-response profile correlated with the absent or decreased isoniazid activity in dormant *M. tuberculosis*. Moreover, this study also showed that the isoniazid-response profile can be found in the early steps of infection in the mouse after isoniazid treatment, but that this response decreases during late-stage infection, correlating with the reduction in isoniazid activity. This may correspond with entry of the bacteria into dormancy at this stage of infection. Isoniazid has no effect in non-replicating bacteria under microaerophilic conditions and the isoniazid-response profile is absent in this condition (Tudo et al., 2010). The authors proposed that the decreased requirement for mycolic acid, modification of the redox state, and cell wall permeability of non-replicating mycobacteria can confer tolerance to isoniazid.

Proteomics approaches provide another means to investigate gene expression and identify changes in protein levels in response to antibiotics. Quantitative proteomics detected increased levels of AcpM, KasA, KasB, and AccD6, proteins involved in the FAS II pathway, after isoniazid treatment, as well as proteins involved in cell division (Hughes et al., 2006). An original study used this approach to compare two isogenic strains of *M. tuberculosis* obtained from the same patient before and after isoniazid treatment (Nieto et al., 2016). Forty-six proteins for which the abundance was altered were identified after the acquisition of isoniazid resistance. The authors emphasized the low levels of KatG found in the isoniazid-resistant strain. Other proteins for which the abundance was modified belong to several categories, in particular, lipid metabolism, intermediate metabolism, and the respiration pathway, especially decreased levels of enzymes of the tricarboxylic acid (TCA) cycle. The authors suggested that these changes may reflect a compensatory mechanism to the reduced amount of KatG.

These observations may also be related to the fact that low metabolic rate contributes to drug tolerance in the host. Several stresses that limit replication, such as low pH or hypoxia, induce triglyceride synthesis and decrease cellular carbon fluxes from the TCA cycle and the accumulation of triacylglycerol (TAG) is associated with decreased growth and increased antibiotic tolerance, both *in vitro* and in the mouse infection model (Baek et al., 2011).

In order to be complete, metabolomic (Zampieri et al., 2018) and lipidomic (Pal et al., 2017) are complementary approaches to study the effect of isoniazid on physiology of mycobacteria. The use of high-throughput mass spectrometry-based lipidomic approach revealed differential lipids profile in MDR compared to drug sensitive *M. tuberculosis* (Pal et al., 2017). Moreover, the composition of different classes of lipids, and not only fatty acyl category, is modified in *M. tuberculosis* after treatment by isoniazid (Pal et al., 2018).

## Transcriptional Signature and Drug Mode of Action

An ambitious study concerning the transcriptional response to antibiotics in *Mycobacterium* consisted of using whole-genome microarrays to measure the effects of 75 different drugs, drug combinations, and growth conditions (Boshoff et al., 2004). The genes that were down- or upregulated at least three-fold were analyzed and grouped by profile, according to coordinately regulated genes. These profiles corresponded to the groups of drugs classified based on their mechanism of action (cell-wall biosynthesis inhibitors, protein synthesis inhibitors, transcriptional inhibitors, or DNA gyrase inhibitors). They corresponded to the signature of a given drug’s mode of action. For example, isoniazid, ethionamide, thiolactomycin, and cerulenin, which inhibit fatty acid and mycolic acid synthesis, displayed a similar expression profile, with induction of the *kas* operon (*acpM-kasA-kasB-accD6*), which encodes an enzyme involved in the FAS II system, and *efpA*, which encodes an efflux pump protein. These profiles were then used to successfully predict the mechanism of action of two previously uncharacterized compounds, phenothiazine, as a respiratory modulator, and pyridoacridone, as an iron scavenger, as well as the mechanism of action of a purified natural product and the product in the crude extract. This method was later applied to other molecules, such as a lipase inhibitor, tetrahydrolipstatin (Waddell et al., 2004), benzothiazinones (Makarov et al., 2009) targeting arabinose metabolism, or pretomanid (PA-824) (Manjunatha et al., 2009), which has a complex mechanism of action that targets both the bacterial cell wall and respiratory complex. Pretomanid is currently being tested in a phase III clinical trial for the treatment of TB and recently, a study using a metabolomic approach, showed the accumulation of a toxic metabolite, a methylglyoxal induced by pretomanid, which participates in the action of the drug (Baptista et al., 2018).

This transcriptional approach has recently been used to compare the response of *M. tuberculosis* and *M. marinum* after treatment with a subinihibitory concentration of ciprofloxacin, ethambutol, isoniazid, streptomycin and rifampicin (Boot et al., 2018) and the authors observe that fingerprint was more distinctive in *M. marinum*. Then, tree functional stress reporters were constructed on the base of stress fingerprint of *M. marinum*, and these reporter strains have made it possible to identify the mode of action of three antimycobacterial compounds previously uncharacterized (Boot et al., 2018).

Transcriptome analysis has also been used to study the effect of natural compounds from plants with antimycobacterial activity, such as chelerythrine, on *M. tuberculosis* (Liang et al., 2011). The authors showed there are many genes that were either up- or downregulated and involved in various pathways, but were unable to propose a mechanism of action of this compound. They noted, however, the up-regulation of genes that encode the 30S and 50S ribosomal proteins, which could be due to an adaptive response or the inhibition of translation by chelerythrine. The genes *hsp, hspX*, and *hspR*, involved in the heat shock response, were downregulated. The decrease in the expression of the gene *Rv0467*, encoding ICL (isocitrate lyase), after chelerythrine treatment is also of interest. Indeed, ICL, a glyoxylate cycle enzyme that converts isocitrate to succinate and glyoxylate, is required for the *in vivo* growth and virulence of *M. tuberculosis* and can mediate broad antibiotic tolerance (Nandakumar et al., 2014). It is a potential drug target for *M. tuberculosis* dormancy (Lee et al., 2015).

On the contrary, ICL has been shown to be induced in *Mycobacterium* by capreomycin (Fu and Shinnick, 2007), a cyclic peptide produced by *Streptomyces capreolus* that inhibits protein biosynthesis in *Mycobacterium* by binding 16S rRNA and 23S rRNA. Capreomycin also induced expression of the genes encoding the 30S and 50S ribosomal proteins, that encode methyltransferase *Rv1988*, and the gene *eis Rv2416c*, which enhance intracellular survival of *Mycobacterium* in macrophages (Fu and Shinnick, 2007). Moreover, most genes specifically induced by capreomycin and PA-824 relative to first-line TB drugs are associated with the stress response and the PE/PPE family expression (Fu and Tai, 2009).

Linezolid is a synthetic antibiotic that belongs to the oxazolidinone class of antimicrobials, which bind to the 50S ribosomal subunits and inhibit protein synthesis. This compound is generally used to treat Gram-positive infections, such as with *Staphylococcus* or *Enterococcus*, but has also been recently used for the treatment of patients with drug-resistant TB (Lee et al., 2012). The transcriptional response to subinhibitory linezolid exposure has been studied in *M. tuberculosis* by microarrays (Liang et al., 2012). The authors reported 729 genes to be differentially regulated by linezolid. They are involved in various pathways, such as protein synthesis, sulfite metabolism, cell-wall permeability, and virulence. Among them, linezolid exposure induced the transcription of chaperone-encoding genes (*dnaK* and *groEL*), which may be a sign of a linezolid-induced stress response. Genes involved in the biosynthesis of sulfolipid-I and TAG were downregulated, whereas genes involved in the synthesis of phthiocerol dimycocerosate (DIM), which plays a role in cell-wall permeability and the virulence of *M. tuberculosis* during macrophage invasion (Astarie-Dequeker et al., 2009), were inhibited. Surprisingly, some of the genes encoding the 50S ribosomal protein were down regulated, whereas others were upregulated in the presence of linezolid. These results are different from those observed for *Staphylococcus aureus*, for which linezolid treatment increased the expression of ribosomal proteins to adjust protein biosynthesis in response to the reduction in translational efficiency (Bonn et al., 2016). Other genes involved in protein synthesis were also down- or upregulated in *M. tuberculosis*. These variations in expression level probably affect protein synthesis rates and could participate in the disturbance of homeostasis by linezolid in Mycobacteria.

The most recent study of the transcriptional response to antibiotics in *Mycobacterium* concerns the response following sublethal kanamycin exposure (Habib et al., 2017). Kanamycin is a second-line drug used for the treatment of patients with MDR-TB. This antibiotic works by binding to the 30S subunit of ribosomes, which interferes with translation and disrupts protein synthesis. Unlike previous studies, in which the authors showed the response to antibiotics to occur relatively early, this study investigated the adaptive response. Indeed, an avirulent strain, *M. tuberculosis* H37Ra, was subcultured every 3 days with a sublethal concentration of the drug to observe transcriptional changes over time. The authors showed that the growth curve of the bacteria cultured in the presence of kanamycin became the same as that of bacteria cultured in the absence of the drug. This corresponds to an adaptation to the presence of kanamycin over time, with the bacteria changing their physiology to adapt to antibiotic stress. Every 3 days, a portion of both the experimental and control cultures were subjected to RNAseq. The authors identified 98 upregulated and 198 downregulated genes in *M. tuberculosis* in the presence of kanamycin. Several upregulated genes belonged to different functional categories, such as cell-wall and cellular processes, virulence, and the PE/PPE family, whereas the downregulated genes were involved in lipid metabolism. Members of the PE/PPE family are proteins associated with the membrane or cell wall of mycobacteria and have been shown to interact with mediators of the host immune response and play a role in pathogenesis (Fishbein et al., 2015) and the genotoxic stress response (Namouchi et al., 2016). Several genes with increased or decreased expression encode uncharacterized proteins. In particular, the upregulated gene *Ra3160* is one of the most responsive genes to kanamycin. Ra3160 and Ra3161 are members of the dormancy regulon (DosR), known to be involved in the survival of bacteria under conditions of stress and antibacterial tolerance (Sivaramakrishnan and de Montellano, 2013). *Ra1750* was strongly induced by kanamycin treatment and was previously shown to be induced during hypoxia through the DosS/DosR system (Sherman et al., 2001; Sivaramakrishnan and de Montellano, 2013). Finally, the authors built gene-interaction clusters which suggest the contribution of these genes to the physiological adaptation of *M. tuberculosis* in response to sub-lethal kanamycin exposure.

Vancomycin is an antibiotic of the glycopeptide family and inhibits peptidoglycan synthesis of the bacterial cell wall, but is considered to have little effect on *Mycobacterium* because of the impermeability of the membrane. However, global transcriptomics detected 153 differentially expressed genes in *M. tuberculosis* after exposure to 10X-MIC vancomycin and 141 differentially expressed genes following exposure to a in subinhibitory concentration of the drug (Provvedi et al., 2009). The high concentration of vancomycin induced the expression of several genes involved in the oxidative-stress response, including the (USP) *rv2623*; chaperone-encoding genes (*hsp* and *htpX*), suggesting that vancomycin causes protein misfolding or aggregation; the toxin-antitoxin (TA) system, which plays a role in the stress response and induction of dormancy; and several genes encoding surface proteins or involved in cell-wall processes. Rv2623 is known to be involved in the regulation of mycobacterial growth and the transition to latency via an ATP-dependent function (Drumm et al., 2009). Thus, induction of the *rv2623* gene, by treatment with vancomycin, could contribute to the induction of persistent infection in the host and difficulty in eradicating the disease. Rv1152 is a transcriptional regulator that belongs to the GntR family and negatively regulates the vancomycin-responsive genes, including *rv2623* (Zeng et al., 2016). The MIC of vancomycin against *M. tuberculosis* is too high to allow its use in the clinic, but the discovery of adjuvants to improve its efficacy, such as a Rv1152 inhibitor, should be envisaged. Drug targeting lipid synthesis, such as that of cerulenin, has also been shown to synergistically increase susceptibility to vancomycin (Soetaert et al., 2015). Moreover, vancomycin treatment induces the transcription of the transcriptional regulator *whiB6*, which is also induced by several types of stress (SDS, ethanol, oxidative stress, heat shock) but slightly downregulated by cycloserine, another inhibitor of cell-wall biosynthesis (Geiman et al., 2006).

Cycloserine, ethambutol, and isoniazid, which all disrupt formation of the cell wall, but by different modes of action, increase the transcription of the transcriptional regulator *whiB2*, whereas the transcription of *whiB7* is increased by protein synthesis inhibitors, such as erythromycin, tetracycline, streptomycin, and kanamycin (Morris et al., 2005; Geiman et al., 2006), and DNA replication inhibitors, such as fluoroquinolones (Burian et al., 2012a). WhiB2 has been reported to act as a chaperone *in vitro* (Konar et al., 2012) and it also an ortholog of the *Streptomyces* sporulation regulators WhiB and it is induced in resting cell formation in *M. smegmatis* (Wu et al., 2016). WhiB7 is a transcriptional regulator induced by exposure to antibiotics and fatty acids of the eukaryotic host (Morris et al., 2005). The *whiB7* promoter can be induced by many diverse compounds, independently of the structure or target of such compounds, and many are respiration and redox homeostasis disrupters (Burian et al., 2012b). Thus, WhiB7 likely regulates redox stress and cell metabolism and likely acts as a transcriptional regulator that coordinates intrinsic drug resistance with antibiotic-induced changes of bacterial physiology (Burian et al., 2012a). A better understanding of the genes regulated by WhiB7 and the identification of compounds that inhibit the products of these genes should provide new insights into the treatment of mycobacterial diseases.

β-lactam antibiotics are a class of broad-spectrum antibiotics that inhibit peptidoglycan synthesis and thus cell wall biosynthesis. However, this class is not usually used for the treatment of TB because *M. tuberculosis* is intrinsically resistant to β-lactam through the production of β-lactamases (Flores et al., 2005). Nonetheless, the association of β-lactam with β-lactamase inhibitors is currently being reconsidered due to the increase in the appearance of MDR-TB (Wivagg et al., 2014; Story-Roller and Lamichhane, 2018).

Meropenem, a member of the carbapenem class of β-lactams, induces the expression of cell-wall biosynthetic genes, such as FbpC (Ag85C), the β-lactamase BlaC *Rv2068c*, and those of the stress pathway, such as Hsp, and membrane proteins, and the multidrug-transport integral membrane protein Mmr (Rv3065); whereas genes involved in various metabolic process are downregulated (Lun et al., 2014).

An analysis of the transcriptional response of *M. tuberculosis* treated with amoxicillin and clavulanate has been performed to clarify the mode of action of the combination of a β-lactam with a β-lactamase inhibitor (Mishra et al., 2017). Such treatment resulted in the upregulation of 481 genes and downregulation of 461; the authors suggest that these modifications represent an adaptation profile that allows tolerance to the combination of drugs. The authors further combined these results with *in silico* network analysis to highlight genes belonging to several functional classes in this transcriptional response. They included cell-wall processes with genes involved in peptidoglycan biogenesis and the induction of genes involved in membrane permeability and the drug efflux pump, as well as those belonging to lipid metabolism, intermediary metabolism, and respiration. Finally, the main finding of this work was a link between tolerance to the combination of the β-lactam and β-lactamase inhibitor with the redox stress mechanism. Indeed, the authors propose that β-lactam affects the integrity of the membrane and thus disturbs the respiratory chain, redox balance, and ATP generation, all which are involved in killing the bacteria. The mycobacteria compensates for these disturbances by modifying its metabolism, which produces reactive oxygen species (ROS). WhiB4, a regulator involved in the oxidative-stress response (Chawla et al., 2012) was also shown to regulate β-lactamase expression associated with redox homeostasis and thus participate in antibiotic tolerance of *M. tuberculosis* (Mishra et al., 2017).

Bedaquiline is a new drug approved for the treatment of MDR-TB since 2012, with the ability to inhibit mycobacterial F1F0 ATP synthase (Andries et al., 2005). Transcriptional analyses revealed that *M. tuberculosis* induces the DosR and activates ATP-generating pathways in the beginning of treatment with to bedaquilin (30 and 180 min) (Koul et al., 2014). The DosR is induced by multiple stresses, especially conditions that inhibit aerobic respiration (Boon and Dick, 2012). Upregulation of the ATP synthase operon could be a strategy to compensate for the inhibition of ATP synthase at the beginning of bedaquiline treatment. A time-course analysis of the transcriptome was performed in *M. smegmatis* to study the bactericidal mode of action of bedaquiline over time and was coupled to the measurement of oxygen consumption and the proton-motive force (PMF) (Hards et al., 2015). Many of the genes upregulated in response to bedaquiline treatment belong to alternative metabolic pathways for energy generation, in particular cytochrome bd oxidase, as well as those involved in central intermediary metabolism and regulatory functions, whereas genes involved in glycolysis, a source of non-respiratory ATP generation, were downregulated. The authors suggested a new mechanism for the bactericidal effects of bedaquiline, at least in *M. smegmatis*, consisting of the uncoupling of respiration-driven ATP synthesis, leading to collapse of the transmembrane pH gradient and dissipation of the PMF (Hards et al., 2015).

Upregulation of the *dosR* regulon and ATP synthase operon is transient given that the expression of these genes is returned to the baseline profile 48–96 h after bedaquiline treatment (Peterson et al., 2016). At these times, the ArsR-type transcription factor Rv0324 and the MarR family transcription regulator Rv0880 have an essential role in the regulation of response to bedaquiline. Moreover, the disruption of these two genes by knocking out makes the bacteria hypersensitive to bedaquiline (Peterson et al., 2016). Rv0324 and Rv0880 would regulate and coordinate the response to bedaquiline treatment that allows the bedaquiline-tolerance state. The authors then managed to demonstrate that pretomanid potentiates killing by bedaquiline via inhibition of the *Rv0880* bedaquiline-response regulon (Peterson et al., 2016).

The compound AX-35 is an arylvinylpiperazine amide that target QcrB subunit of the respiratory cytochrome bc_1_ complex (Ballell et al., 2013; Foo et al., 2018). The treatment of *M. tuberculosis* by AX-35 causes the upregulation of the *cydABCD* operon, but not the *dosR* regulon, which is compatible with the signature profile of compounds inhibiting cytochrome c oxydase (Boshoff et al., 2004). *cydA* and *cydB* are the structural genes coding the cytochrome *bd* terminal oxidase while *cydCD* encodes an ABC transporter implicated in cytochrome *bd* assembly. The authors also note an upregulation of *tgs* genes and lipases genes (*lipU* and *lipR*) involved in the biosynthesis of TAG which would correspond to a remodeling of the central carbon metabolism and lipid metabolism induced by AX stress in *M. tuberculosis* (Foo et al., 2018).

## DNA Repair Pathway Induced by Subinhibitory Concentrations of Ciprofloxacin

It is known that bacterial stress causes various adaptive and protective responses and modifies gene expression patterns and cell physiology, which can influence susceptibility to antibiotics (Poole, 2012). However, addition of an antibiotic can itself be considered to be a stress. The effect of antibiotics on bacteria is not only target-specific inhibition. Bactericidal antibiotics can induce the production of toxic metabolites that lead to cellular damage (Belenky et al., 2015). Antibiotics, such as β-lactams, quinolones, and aminoglycosides, can induce the production of ROS in bacteria (Kohanski et al., 2007). In addition, several studies involving different antibiotics and different species of bacteria have shown that ROS cause damage to macromolecules, including DNA, which can result in induction of the SOS response and thus SOS-induced mutagenesis, leading to the appearance of resistance (Dwyer et al., 2009; Kohanski et al., 2010a).

Fluoroquinolones have antimycobacterial activity and are used for the treatment of TB as second-line drugs. They prevent cell division by inhibiting DNA gyrase, which belongs to the type II topoisomerase family, thus preventing the replication of bacterial DNA. In addition, inhibition of DNA gyrase results in the appearance of double-stranded DNA breaks, inducing the SOS response. The SOS system is an error-prone repair system which can induce mutations. Gyrase inhibitors also induce the formation of ROS and oxidative damage (Dwyer et al., 2007; Kohanski et al., 2010a), which can also lead to mutagenesis. The contribution of these mutations to adaptation or antibiotic resistance is still a subject of debate (Kohanski et al., 2010b; Torres-Barcelo et al., 2015). A study in *Mycobacterium* showed that a subinhibitory concentration of ciprofloxacin, which belongs to the fluoroquinolone class, increased mutation rates (Gillespie et al., 2005). It is in this context that a study was performed to investigate the effects of the response of *M. tuberculosis* to subinhibitory concentrations of ciprofloxacin by genome-wide expression profiling and qRT-PCR (O’Sullivan et al., 2008). The microarray analysis showed upregulation of 16 genes involved in DNA protection, repair, and recombination, including *recA*, known to be involved in the SOS response and stimulated by single-stranded DNA. Differential expression of *lexA* and *dnaE2*, two other components of the SOS response, was not observed by microarray analysis, but qRT-PCR showed an increase in the expression of these two genes. DnaE2 polymerase is known to contribute to the emergence of drug resistance in *M. tuberculosis* (Boshoff et al., 2003). It is thus important to extend this research to investigate the possibility of inducing resistance by fluoroquinolone in the treatment of TB.

The transcriptional response to ciprofloxacin has also been studied in *S. coelicolor* (Patkari and Mehra, 2013), a non-pathogenic soil bacterium belonging to the Actinobacteria phylum, the same as *Mycobacterium*. The exposure of *Streptomyces* to ciprofloxacin induced the transcription of the gene encoding the target, DNA gyrase, as well as genes involved in DNA repair and various transporters which could function as efflux pumps. The authors also noted the production of ROS, as well as the induction of SOS genes by ciprofloxacin. Thus, bacteria may increase the transcription of DNA repair and SOS genes to repair damaged DNA as a consequence of ciprofloxacin treatment.

## The Transcriptomic Response to Antibiotics During Infection

Most data on the mode of action of antibiotics have been obtained using cultures under laboratory growth conditions and the studies presented in this review up to now have focused on the *in vitro* effects of drugs against *Mycobacterium*. However, these approaches may not reflect reality *in vivo*, in humans infected by mycobacteria or animal models of infection. It is thus important to explore the transcriptional response *in vivo* during the treatment of TB.

Several studies have investigated the transcriptional response of *Mycobacterium* inside macrophages, providing new insights into the adaptation of the mycobacteria in the cellular environment and changes in their metabolism during progression of the disease (Waddell and Butcher, 2007). The absence of induction of isoniazide-responsive genes after isoniazide treatment in an animal model and patients suggests that the mycobacteria have become tolerant to isoniazid (Karakousis et al., 2008; Walter et al., 2015). Thus, clinically, the analysis of the antibiotic-response by transcriptomic profiling should make it possible to predict the physiological state of the bacteria and the induction of antibiotic tolerance. Such information could contribute to improving treatment strategies.

Gene expression has been investigated in samples from patients with pulmonary drug susceptible TB treated with the standard combination of isoniazid, rifampicin, pyrazinamide, and ethambutol (Walter et al., 2015). A decline in bacterial mRNA abundance showed killing of most of the bacteria 4 days after starting treatment, but the abundance of bacterial mRNA after that indicates drug tolerance. Genes involved in growth, metabolism, and lipid synthesis were downregulated in the drug-tolerant bacteria, whereas those involved in the stress response and drug efflux pumps were upregulated. In particular, the upregulation of regulatory genes, such as those of the toxin-antitoxin (TA) module, sigma factors, and transcription factors were observed, as in previous transcriptomic studies of persister cells (Betts et al., 2002, 2003; Muttucumaru et al., 2004). Moreover, the transcriptional signature of isoniazid stress was induced at the beginning of treatment but disappeared after 4 days. This suggests that the response to antibiotics of drug-tolerant *M. tuberculosis in vivo* changes, as observed in drug-treated dormant *M. tuberculosis* (Karakousis et al., 2008).

## The Transcriptomic Response in Clinical Strains and Multidrug Resistant Strains

Global expression profiling studies have also been carried out to investigate the response of resistant clinical strains to antibiotics. Some genes appear to be differentially expressed between sensitive H37Rv strain and drug-resistant strains (Penuelas-Urquides et al., 2013; Yu et al., 2015). Thus, *esxG, esxH, rpsA, esxI*, and *rpmI* have been shown to be upregulated, whereas *lipF, groES*, and *narG* were downregulated in MDR clinical strains (Penuelas-Urquides et al., 2013). However, the expression of these genes among different clinical strains with similar drug-resistance profiles varies widely (Gonzalez-Escalante et al., 2015). EsxG-EsxH are proteins secreted by the ESX-3 type VII secretion system required for optimal growth and may play a role in the virulence of *M. tuberculosis* (Ilghari et al., 2011; Tufariello et al., 2016). Nine intergenic regions were also upregulated in the MDR strain (Penuelas-Urquides et al., 2013). Whether these intergenic regions code for proteins or are non-coding RNA with a role in post-transcriptional regulation is yet to be determined (Arnvig et al., 2011).

A transcriptional profiling study to assess the accumulation of drug resistance in the Mumbai area showed genes for cell-wall biosynthesis (*emb*), protein synthesis (*rpl*), transcription factors (*sig, rpoB*), and those involved in various metabolic pathways to be downregulated, whereas genes encoding efflux pumps and transcriptional factors involved in the stress response (*whiB*) where upregulated in MDR clinical isolates relative to those in a drug-susceptible strain (Chatterjee et al., 2013). Such differences could contribute to resistance to anti-TB drugs, without the presence of drug-resistance mutations. The upregulation of efflux pumps allows expulsion of the antibiotics whereas the decrease in cellular metabolism and transcription could be associated with a form of persistence.

Another comparison of the transcriptome of XDR clinical isolates and susceptible *M. tuberculosis* revealed downregulation of *ethA* (de Welzen et al., 2017), which encodes the monooxygenase that activates ethionamide. Such downregulation was due to a mutation in the promoter, but not transcriptional repression by *ethR*, and the strains with this mutation were resistant to ethionamide without other known ethionamide resistance-determining genotypes.

Transcriptional responses to isoniazid, capreomycin, and rifampicin have been investigated in two XDR strains compared to that of H37Rv, a wildtype strain (Yu et al., 2015). The authors highlighted a correlation between genes overexpressed in the XDR strains and previously published known drug resistance genes and proposed 92 as candidate resistance genes. Regulatory network analysis of these genes showed the importance of the transcriptional regulator CRP (Rv3676) and three two-component regulatory systems (TCS) in drug resistance: SenX3-RegX3, MprA-MprB, and MtrA-MtrB. TCS are involved in sensing the response to stress and adaptation to changes in the environment (Bretl et al., 2011; Parish, 2014) and the coordination of global changes in gene expression. It is interesting to note that SenX3 and MtrA share homology with the gene expression modulator BfmRS, which has recently been shown to enhance pathogenicity and antibiotic resistance in *Acinetobacter baumannii* (Geisinger et al., 2018).

Rifampicin is a first line TB drug that binds to the pocket of the RNA polymerase β subunit (RpoB) and thus inhibits RNA synthesis. In 95% of cases, *M. tuberculosis* becomes resistant to rifampicin by target alteration after mutation of the gene *rpoB*. A transcriptional profiling study was performed using microarray technology on wildtype rifampicin-susceptible and rifampicin-resistant strains exposed to several concentrations of rifampicin to obtain new insights into the response to rifampicin exposure (de Knegt et al., 2013). The transcriptional response induced by rifampicin was different between the rifampicin-resistant and rifampicin-susceptible strains. In particular, the authors highlighted changes in the expression of gene clusters associated with efflux, transport, and virulence. Moreover, the authors observed strong upregulation of both genes of operon *Rv0559c-Rv0560c*, previously shown to be induced following salicylate exposure (Denkin et al., 2005) and recently shown to be upregulated upon isoniazide exposure (Gamngoen et al., 2018). The mechanism and function of Rv0560c and Rv0559c in response to two of the first-line TB drugs, rifampicin and isoniazid, merit further study.

The mutation in *rpoB* can be associated with a fitness cost for bacteria (Mariam et al., 2004; Gagneux et al., 2006) but it has been proposed that mutation in genes *rpoC* could compensate for this fitness cost (Comas et al., 2011). Transcriptional approach seems to confirm this proposition by comparing responses between clinical strains that harbor single mutation in *rpoB* and clinical strains with mutations in *rpoB* and *rpoC* (Xu et al., 2018). The energy metabolism and oxidative phosphorylation machinery are disrupted in the *rpoB* single mutants but the upregulation of the bd-type menaquinol oxydase (CydAB) and of *qcrCAB* operon, encoding cytochrome c oxydase, in *rpoBC* mutants might compensate this phenomenon. *rpoC* mutations were also associated with upregulation of ribosomal protein genes and alteration in amino acid synthesis pathways, these changes in metabolism may participate in the compensatory response (Xu et al., 2018).

It is well known that the multidrug efflux pumps contribute to antibiotic resistance and/or tolerance, as well as bacterial virulence (Alcalde-Rico et al., 2016). Efflux pumps can be expressed constitutively and participate in intrinsic resistance, or their expression can be stimulated by an effector and thus result in phenotypic resistance to an antibiotic. Various efflux pump genes can be up-regulated in the presence of many antibiotics, as already discussed. An exhaustive study underlined the importance of this mechanism of drug resistance by comparing the differential expression of efflux pump genes in the presence of various antimycobacterial drugs in several clinical MDR isolates of *M. tuberculosis* (Gupta et al., 2010). This study highlighted the association between differential expression of selective efflux pump genes and drugs, such as ethambutol, isoniazid, and streptomycin, as well as strain-to-strain variations. The same phenomena was observed for the overexpression of efflux pump genes by isoniazid and rifampicin in MDR isolates (Li et al., 2015) and the basal expression level of some genes encoding efflux pumps was higher in MDR than sensitive isolates.

## Discussion

Antibiotic-induced changes in gene expression profiles have aided our understanding of the effect of antibiotics on the physiology of *M. tuberculosis*. The investigation of such changes, measured by microarrays or RNA-seq, can help to identify the mode of action of the drug tested, as well as provide a better vision of the mechanisms of drug tolerance or drug resistance. The genes that promote antibiotic resistance have collectively been called the resistome (Wright, 2007).

The gene expression profile induced by a drug can be considered to be the transcriptional signature characteristic of its mode of action and this signature can be used to predict the mode of action of novel compounds with antimycobacterial activity, identified by whole cell-screening approaches. However, the quality of the expression data is essential to predicting the mode of action of new drugs based on comparisons with the profile induced by well-characterized antimycobacterial compounds, as shown in the study of Boshoff et al. (2004).

It may be difficult to directly compare the presented studies because of the different methods used. The modulation of mycobacterial gene expression depends on not only the class of antibiotic but also the concentration tested. Antibiotics can be tested at high concentrations, equivalent to or higher than the MIC that prevents growth of the bacteria, or at a subinhibitory concentration to explore the physiological adaptation of the bacteria. The duration of exposure to the antibiotic should also be taken into consideration.

In addition to having an effect on a specific target, an antibiotic can affect the global physiology of the bacteria. In transcriptomic studies, it can be difficult to discriminate between changes in RNA expression corresponding to compensation for inhibition of the target pathway by the drug and those that are a response to the toxic effect of the compound. Indeed, the changes in gene expression in response to an antibiotic are a combination of the specific effect of the drug and a global metabolic effect that involves the deregulation of many genes (Mitosch and Bollenbach, 2014). For example, the activity of transcriptional regulators and RNA polymerase can be modified upon antibiotic treatment and thus influence the expression of all bacterial genes. Moreover, antibiotics affect the growth rate of bacteria and it has been shown that changes in bacterial growth rates modify gene expression in bacteria in chemostat culture (Hua et al., 2004).

However, the comparative analysis of transcriptome data from *Mycobacterium* treated with different antimycobacterial drugs can be used to predict new drug combination. From these comparisons, After having identified Rv0324 and Rv0880 as regulators of bedaquiline tolerance in *M. tuberculosis*, pretomanid has been identified as a modulator of expression of *Rv0880* and a potentiator of bedaquiline activity (Peterson et al., 2016).

We have seen that the transcriptomic data can be used to identify new antibacterial compounds. Genes induced specifically by one class of antibacterial can be used as reporter to identify compounds sharing the same mode of action. In order to identify cell envelope inhibitor, a synthetic compounds library was screened for induction of *iniBAC* promoter, a marker of cell envelope stress response (Shetty et al., 2018). This approach has identified new acetamide that indirectly targets the essential mycolic acid transporter MmpL3 by disruption of the proton motive force (PMF) that MmpL3 use for translocation to translocate trehalose monomycolates (THM) (Shetty et al., 2018).

The measurement of the variation of mRNA expression is only an indication of the phenotype of bacteria. Metabolomics approaches, which measure small molecules produced during the metabolism, directly reflect the biochemical activity of bacteria. Changes in the metabolome profiling after treatment with antimicrobial compounds give new insight in the bacterial physiological response to antibiotic and the drug mechanism (Zampieri et al., 2017) and this analysis have been used in *M. smegmatis* to predict the mode of action of uncharacterized antimicrobial compounds (Zampieri et al., 2018).

The environmental context of *M. tuberculosis* affects mRNA expression, as the regulation of gene expression also depends on the host microenvironment. Once inside macrophages, the bacteria must adapt to the intracellular environment and accommodate the host immune response. Upregulated genes of *M. tuberculosis* inside macrophages include those involved in fatty acid metabolism, mycolic acid modification, the DosR (Schnappinger et al., 2003), and several members of the WhiB family (Rohde et al., 2007). Thus, the transcriptional response of bacteria to antibiotic exposure *in vitro*, in the laboratory, is likely to be very different from that in the intracellular context.

A study showed the role of host macrophages in the induction of efflux pumps and drug tolerance of Mycobacteria (Adams et al., 2011) using a zebrafish model of TB infection with *M. marinum*. The study showed that Rv1258c, a member of the MFS of efflux pumps, which is transcriptionally induced after entry into the macrophage (Schnappinger et al., 2003), promotes intracellular growth in macrophages in the absence of drug treatment, as well as intracellular rifampicin tolerance during treatment (Adams et al., 2011).

The metabolism of mycobacterial cells can change during the dynamics of the infection, in particular when the bacilli enter into the dormant state, in which metabolic activity is reduced, leading to antibiotic tolerance or resistance. Persister mycobacteria are phenotypically resistant to antimycobacterial compounds, although genetically susceptible to these drugs (Gengenbacher and Kaufmann, 2012). Thus, the phenotypic plasticity of mycobacteria allows them to survive in the presence of antibiotics (Ehrt et al., 2018).

After to have identified a set of genes induced in *M. tuberculosis* persister cells surviving 4 days of isoniazide treatment, the promoter of these genes was fused with fluorescent reporters in mycobacteriophages. This tool helped to identify a population of preexisting persister mycobacteria in the sputum of human patients and the number of these persister cells increased after the beginning of antitubercular therapy (Jain et al., 2016).

In addition, factors of variability inherent to the host, such as the genetic background or the efficiency of the host’s immune response, can also influence the bacterial transcriptional response, as recently shown for *S. aureus* (Thanert et al., 2017). It has been observed that susceptibility to TB varies according to genetic predisposition among populations (Cooke and Hill, 2001; Thye et al., 2010). Moreover, patient responses to treatment are heterogenic; transcriptomic and metabolomic profiles of patients infected by *M. tuberculosis* were similar before treatment, but different after treatment (Tientcheu et al., 2015). Thus, human polymorphism also influences the behavior of the bacteria. Given these considerations, the next major challenge will be the exploration of gene expression changes induced by antibiotics during *in vivo* infection.

*Mycobacterium tuberculosis*, possesses genes that can be considered to be drug-resistance systems through modulation of their expression, but the interaction between the drug, the bacteria, and the host is complex. Bacteria can change their global gene expression to increase their chances of survival. Gene regulatory networks appear to be important for preserving cell homeostasis under conditions of stress, such as antibiotic exposure. Transcriptional profiles and phenotypic responses of bacteria under stress are generally highly correlated. Phenotypic and transcriptional stress responses can involve distinct gene sets (Jensen et al., 2017). It has been shown in *Streptococcus pneumonia* that nutrient stress causes a highly organized response, whereas the response to antibiotics is uncoordinated (Jensen et al., 2017). The construction of transcriptomic data sets, complemented with proteomic (Wenzel and Bandow, 2011), metabolomic data (Zampieri et al., 2018), and lipidomic (Pal et al., 2017) should bring new insights into the molecular networks involved in the response to antibiotic exposure.

Deregulated genes identified after antibiotic exposure can be considered to be targets for the identification of new drugs and the development of therapy against TB. These drugs, which would target potential resistant pathways, could be used in combinatorial therapies with existing anti-TB drugs. Antibiotic adjuvants or potentiators are compounds that enhance antibiotic activity by inactivating resistance mechanism (Wright, 2016). As a proof of concept, it was shown that the MIC of isoniazid decreased in the presence of various categories of efflux pump inhibitors in clinical isolates of *M. tuberculosis* (Jaiswal et al., 2017). Efflux pump inhibitors should thus be considered to improve current TB therapy (Pule et al., 2016).

## Author Contributions

JB, SL, and BG have decided the content of the manuscript. JB has analyzed the bibliography and written the manuscript. BG and SL has corrected and approved the manuscript.

## Conflict of Interest Statement

The authors declare that the research was conducted in the absence of any commercial or financial relationships that could be construed as a potential conflict of interest.
